# Soil Moisture Retrival Based on Sentinel-1 Imagery under Sparse Vegetation Coverage

**DOI:** 10.3390/s19030589

**Published:** 2019-01-30

**Authors:** Shuai Huang, Jianli Ding, Jie Zou, Bohua Liu, Junyong Zhang, Wenqian Chen

**Affiliations:** 1 College of Resources and Environment Sciences, Xinjiang University, Urumqi 830046, China; 107556516063@stu.xju.edu.cn (S.H.); 107556515056@stu.xju.edu.cn (J.Z.); 107556517070@stu.xju.edu.cn (B.L.); 107556516060@stu.xju.edu.cn (J.Z.); 107556516059@stu.xju.edu.cn (W.C.); 2 Key Laboratory of Oasis Ecology under Ministry of Education, Xinjiang University, Urumqi 830046, China

**Keywords:** microwave remote sensing, Sentinel-1, AIEM model, soil moisture

## Abstract

Soil moisture is an important aspect of heat transfer process and energy exchange between land-atmosphere systems, and it is a key link to the surface and groundwater circulation and land carbon cycles. In this study, according to the characteristics of the study area, an advanced integral equation model was used for numerical simulation analysis to establish a database of surface microwave scattering characteristics under sparse vegetation cover. Thus, a soil moisture retrieval model suitable for arid area was constructed. The results were as follows: (1) The response of the backscattering coefficient to soil moisture and associated surface roughness is significantly and logarithmically correlated under different incidence angles and polarization modes, and, a database of microwave scattering characteristics of arid soil surface under sparse vegetation cover was established. (2) According to the Sentinel-1 radar system parameters, a model for retrieving spatial distribution information of soil moisture was constructed; the soil moisture content information was extracted, and the results were consistent with the spatial distribution characteristics of soil moisture in the same period in the research area. (3) For the 0–10 cm surface soil moisture, the correlation coefficient between the simulated value and the measured value reached 0.8488, which means that the developed retrieval model has applicability to derive surface soil moisture in the oasis region of arid regions. This study can provide method for real-time and large-scale detection of soil moisture content in arid areas.

## 1. Introduction

In the Earth system, surface soil moisture is an important factor in the process of energy exchange between the land and atmosphere and has a strong control effect on land surface evapotranspiration, water transport, and the carbon cycle [1,2]. As an important component of the water cycle of terrestrial ecosystems [3], soil moisture is the basic condition for plant growth and development, as well as an important indicator for studying plant water stress, monitoring drought conditions, and estimating crop yields. Therefore, accurate monitoring of soil moisture over a large area has great significance in the fields of hydrology, meteorology and agricultural science [4]. In China, the proportion of rain-fed farming in agriculture is quite large [5], and soil moisture is an important indicator to characterize agricultural drought [6], which has important guiding significance for agricultural drought monitoring and early warning [7,8]. In some arid and semi-arid areas, such as Xinjiang, soil salinization has become an important land degradation factor that threatens the local ecological environment, hindering agricultural production and socioeconomic development in these areas. The occurrence of soil salinization is closely related to soil moisture; therefore, the dynamic monitoring of soil moisture can provide corresponding measures and countermeasures for the monitoring and prediction of soil salinization and ecological regulation in these areas.

Wide-ranging monitoring of soil moisture is a recognized problem [9,10]. Compared with traditional in situ measurement methods, remote sensing extends the “point” measurement method that can obtain limited representative information into objective real “zone” information, thereby enabling regional-scale soil moisture information acquisition [11]. Studies have shown that optical remote sensing is susceptible to cloud and other weather conditions and have weak penetrability, while passive microwaves remote sensing have low resolution and long revisit period, which active microwave can make up for the shortcomings of other methods in soil moisture monitoring to improve the reliability and accuracy of soil moisture inversion [12]. This process could provide promising approaches for large-area, real-time soil moisture monitoring.

Over the past few decades, active microwave remote sensing data have been successfully used for estimating soil moisture due to a finer spatial resolution [13]. Several soil moisture models have been proposed to the bare land, including the statistical models (Oh model [14] and Dubois model [15]) and physical models (the integral equation model (IEM) [16] and advanced IEM (AIEM) [17,18,19,20,21]). These models, can only be used in bare land, combined with vegetation scattering models, can derive the soil moisture over vegetated areas. The most widely used vegetation scattering model is the Water-cloud Model [22,23]. Based on this, several soil moisture retrieval algorithms have been developed and tested for multiple SAR satellites operated at the L/C/X-bands [24], such as ALOS-2 [25,26], Radarsat-2 [27,28], ASAR [29], Sentinel-1 [30,31,32], and TerraSAR-X [33], studies that have used these data to estimate bare soil moisture have achieved promising results. With shorter revisit time, the Sentinel-1 mission is expected to reduce the ill-posed retrieving using the time-series data. Up to now, Sentinel-1 has shown its potential on soil moisture retrieval, while the retrieving performances still need more evaluations [34].

The radar backscattering coefficient is affected by the radar’s system parameters and other surface parameters (the dielectric constant, vegetation layer and surface roughness) [35]. The soil dielectric constant is the dielectric property of the soil, which depends on soil texture, soil moisture, frequency etc. Small changes in the soil moisture content can largely change the complex permittivity of the soil, thereby affecting the backscattering coefficient of active microwave observation, which is the theoretical basis for microwave remote sensing to extract soil moisture information [36]. Therefore, the main problem in retrieving soil moisture is eliminating the influence of surface roughness and vegetation cover attenuation and scattering to determine the relationship between backscatter coefficient and soil moisture. It is necessary to understand the relationship between the backscatter coefficient and various parameters. According to these relationships, the optimal radar operating parameters are selected, the surface roughness and vegetation effects are minimized, and the sensitivity of the backscattering coefficient to soil moisture is improved.

This paper takes the Ugan-Kuqa River Delta Oasis as the target area and uses the Advanced Integrated Equation Model (AIEM) model to simulate the surface scattering characteristics, to establish the bare surface, and to determine the soil moisture inversion algorithm and inversion of the spatial distribution of soil moisture in the study area.

## 2. Study Area and Data

### 2.1. Overview of the Study Region

**Figure 1:**
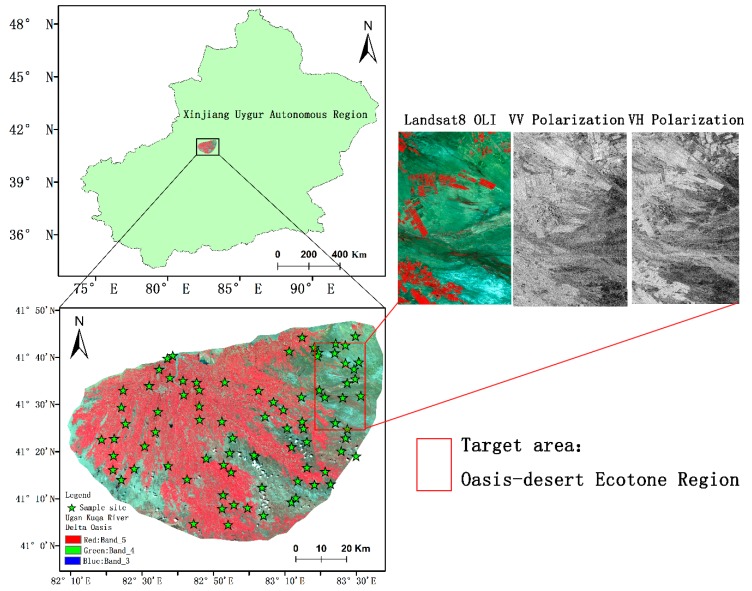
Geographical location of the study area and the field monitoring sites.

In this study, we modeled the Ugan-Kuqa River Delta Oasis(latitude 41°0′ N–41°5′ N, longtitude 82°10′ E–83°30′ E), located on the northern edge of the Tarim Basin in Xinjiang; Luntai County lies to the east, Wensu County lies to the west, the north bank of the Tarim River lies to the south, and the Qiulitage Mountain in the southern foothills of the Tianshan Mountains lies to the north (Figure 1). It is a typical fan-shaped plain oasis in China. The oasis has temperate continental arid climate with abundant light and heat resources. The climate is very dry with a deficiency of rainfall. To be specific, the annual total radiation is 6.11 × 10^5^ J cm^2^, the annual sunshine hours are approximately 2.85 × 10^3^ h, the sunshine rate is 65%, the annual average temperature is 11.3 °C, the potential evaporation is 2356 mm, the average annual precipitation is 55.45 mm, and the drying index is 42. The oasis agriculture is mainly based on planting. Artificial vegetation mainly includes cotton, corn and winter wheat, while natural vegetation is mainly composed of *Tamarix chinensis* Lour, *Halocnermum* str, *Karelinia caspia* (Pall.) Less and *Haloxylon ammodendron* (C. A. Mey.) Bunge [37]. The Ugan-Kuqa River Delta Oasis Interlaced Zone was selected as the target area because the vegetation coverage of this typical plot is sparse, and the surface fluctuations do not change much.

### 2.2. Observation Data

The soil sample sites were collected at a depth of 5 cm in July 2017, coinciding with the Sentinel-1 imagery acquisition times that were used. We used a random clustered sampling technique to allocate 94 sample sites in the Ugan-Kuqa River Delta Oasis. Each sample site should be representative of the soil surrounding a larger area, which contained five points and had a minimum distance of 2 km to the next site to avoid autocorrelation, while the sites were selected away the trails and consisted of homogenous land use types (Table 1). The landscape in this region includes farms, rivers, wetlands, grassland, and bare soil surfaces. Soil data process was divided into two steps:

(1) The soil sample of each site was uniformly mixed and placed into an aluminum box, which would be weighed and recorded, then transported back to the laboratory for drying until the soil was completely dehydrated, cooled to room temperature and weighed in a cool place, the soil moisture was calculated
(2) The collected soil samples were transported to the laboratory, the plant and gravel impurities were removed after air drying, the soil was ground and sieved with a 0.5 mm aperture sieve, and then, physical and chemical analyses were conducted to measure soil texture and soil bulk density.

### 2.3. Satellite Data

#### 2.3.1. Sentinel-1 Data

With all-weather imaging capabilities, Sentinel-1 provides medium and high resolution terrestrial, coastal, and sea ice measurements with single and dual polarization, short revisit cycles, and strong interference capabilities for global high-resolution monitoring. It also provides technical support for long-term sequence soil moisture monitoring in the same area [38]. The data were downloaded from https://scihub.copernicus.eu/dhus/#/home.

The microwave data source used in this study is the dual-polarization (including VV and VH polarization modes) Sentinel-1 data. The data was acquired on 15 July 2017, which with the so-called interferometric wide swath mode at a spatial resolution of 5 m by 20 m. The azimuth width is 250 km. The Sentinel-1 image of the study area is an ellipsoid geocoded Ground Range Detected (GRDH), preprocessed by the SARscape 5.2.1^®^ module developed by SARmap in ENVI 5.3^®^ software. Following these procedures, the processed SAR data with a 20 m spatial resolution are projected onto WGS 1984 Universal Transverse Mercator (UTM) coordinates. The preprocessing of Sentinel-1 data are as follows:

(1) Multi-Look processing (generating power images) makes the image texture structure close to the real situation and reduces the speckle noise.
(2) Filtering and denoising processing (refined-Lee filtering, 3 pixels by 3 pixel window) were performed to eliminate speckle noise.
(3) Geocoding was performed, using digital elevation maps for geometric fine correction.
(4) Radiation calibration was conducted to obtain the backscattering coefficient in the multipolarization mode of the target region.

#### 2.3.2. Landsat-8 OLI Data

Landsat-8,which was launched in February 2013 by NASA, carries two sensors, the OLI Land Imager and the TIRS Thermal Infrared Sensor. For this study, OLI data from July 15, 2017 were downloaded from https://earthexplorer.usgs.gov/. The preprocessing of Landsat-8 OLI data includes radiation correction and atmospheric correction, which are conducted using the ENVI 5.3^®^ software (Exelis Visual Information Solutions, Broomfield, CO, United States). Following these procedures, the processed OLI data with a 30 m spatial resolution are also projected onto WGS 1984 UTM coordinates. NDMI layer was created using band 5 (865 nm) and band 7 (2200 nm) using NDMI processor in ENVI. Furthermore, *vwc* layer was created using NDMI layer using band math processor in ENVI.

## 3. Methodology

**Figure 2:**
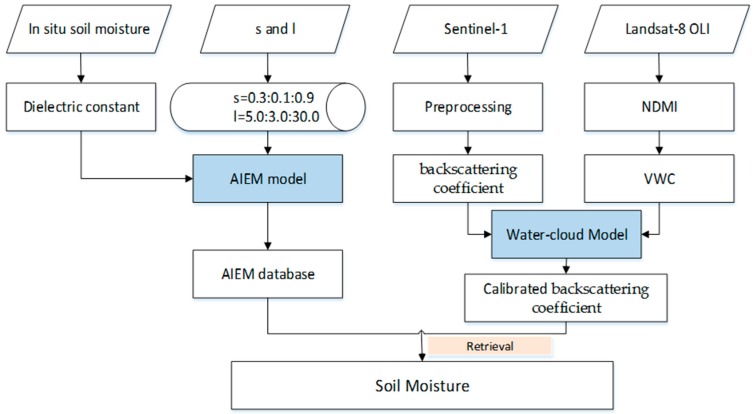
Flowchart for the soil moisture retrieval.

In this paper, the Water-cloud Model and the AIEM were used to estimate the soil moisture in the Ugan-Kuqa River Delta Oasis. The Water-cloud Model is used to simulate the vegetation backscattering, and the AIEM is used to simulate the bare soil backscattering. The implementation of the two models soil moisture retrieval can be seen in Figure 2, and detailed descriptions of the two models are introduced in the following sections.

### 3.1. Water-Cloud Model

The backscattering coefficient obtained from the soil surface of saline soil covered by vegetation is affected by the roughness, soil moisture, salt content and vegetation. Therefore, to accurately extract the radar backscattering characteristics of the soil surface, the vegetation effect should be removed. In the actual situation of the surface coverage of the radar area, the sparse vegetation coverage area is larger than the farmland area. Therefore, the semi-empirical water-cloud model [39] was selected as the vegetation impact correction model. The water-cloud model contains two hypotheses: the vegetation layer is uniform and similar in size and shape, and it ignores multiple scattering between vegetation and the Earth’s surface. The water-cloud model can be expressed as follows:

(1) $$\sigma_{con}^{0}\left(\theta \right)=\sigma_{veg}^{0}\left(\theta \right)+\gamma^{2}\sigma_{soil}^{0}\left(\theta \right)$$

(2) $$\sigma_{veg}^{0}\left(\theta \right)=A\cdot vwc\cdot \cos\left(\theta \right)\left[1-\gamma^{2}\left(\theta \right)\right]$$

(3) $$\gamma^{2}\left(\theta \right)=exp\left[-2B\cdot \frac{vwc}{\cos\left(\theta \right)}\right]$$

In the equations, $\sigma_{con}^{0}\left(\theta \right)$ is the backscattering coefficient of the underlying surface radar, $\sigma_{veg}^{0}\left(\theta \right)$ is the backscattering coefficient of the vegetation layer radar, $\gamma^{2}\sigma_{soil}^{0}\left(\theta \right)$ is the two-way vegetation radar backscattering coefficient of the soil surface after attenuation, $\gamma^{2}\left(\theta \right)$ is the double-layer attenuation factor of the radar band penetrating the vegetation layer, *vwc* is the average water content of all vegetation in the pixel, and A and B are the correction values of vegetation moisture content parameters of different underlying surfaces [40] (Table 2). Based on the actual situation and research focus of the study, the empirical parameters of all vegetation equations were selected, namely A = 0.0009, B = 0.032 [41].

The influence of vegetation on radar backscattering coefficient is shown in Equation (4), and Equation (5) is the backscattering coefficient of the soil after removing the vegetation effect:

(4) $$\sigma_{veg}^{0}\left(\theta \right)=0.0009\cdot vwc\cdot \cos\theta \left[1-exp\left(-0.064\cdot vwc/\cos\theta \right)\right]$$

(5) $$\sigma_{soil}^{0}\left(\theta \right)=\frac{\sigma_{con}^{0}\left(\theta \right)-0.0009\cos\theta \left[1-exp\left(-0.064\cdot vwc/\cos\theta \right)\right]}{exp\left(-0.064\cdot vwc/\cos\theta \right)}$$

### 3.2. Advanced Integral Equation Model

The Advanced Integral Equation Model was proposed by Chen et al. in 2003 based on the Integral Equation Model (IEM) [21], developed in 1992 by Fung et al. [16]. AIEM is more accurate and more widely applicable than the Kirchhoff model because it adds a compensation term to the Kirchhoff model. Therefore, whether the compensation term is accurate or not critically affects the accuracy of the IEM model. In the original IEM derivation, the Green’s function in the compensation field coefficient and the phase in the gradient are simplified.

To calculate the compensation field coefficient more accurately, AIEM re-derived the remnant field strength coefficient and kept the absolute phase term of the Green’s function and its gradient during the derivation, to deduce a more complete and accurate multiple scattering expression and single secondary scattering term. In addition, in calculating the two-station scattering coefficient of a random dielectric surface, the Fresnel reflection coefficient is usually assumed to be approximated by an incident angle or a zero degree angle in low and high frequency ranges. However, both approximations can only be applied to their respective valid ranges. These two approximations can be joined by a transition function, which is included in the AIEM model. The introduction of the absolute phase term of the Green’s function and its gradient and introduction of the transition function during the derivation are two major improvements of AIEM [16,19,42,43,44].

The form of AIEM is more complicated than the IEM description but is still algebraic; thus, it is faster to calculate and achieve better accuracy.

The AIEM model expression is as follows:

(6) $$\sigma_{pq}^{0}=\frac{{k_{i}}^{2}}{2}e^{-s^{2}\left(k_{z}^{2}+k_{sz}^{2}\right)}\sum_{n=1}^{\infty }s^{2n}{\left|I_{pq}^{n}\right|}^{2}\frac{W^{n}\left(k_{sx}-k_{x},k_{sy}-k_{y}\right)}{n!}$$

(7) $$I_{pq}^{n}={\left(k_{sz}+k_{z}\right)}^{n}f_{pq}e^{-s^{2}k_{z}k_{sz}}+\frac{k_{sz}^{n}(F_{pq}\left(-k_{x},-k_{y}\right)+k_{z}^{n}F_{pq}\left(-k_{sx},-k_{sy}\right)}{2}$$

where pq is the polarization mode, $k_{i}$ is the spatial free wavenumber, δ is the root mean square height, $W^{n}\left(k_{sx}-k_{x},k_{sy}-k_{y}\right)$ is the Fourier transform of the surface correlation function, $k_{z}=k\cos\theta_{i}$, $k_{sz}=k\sin\theta_{s}$, $k_{x}=k\sin\theta_{i}\cos\varphi$, $k_{sx}=k\sin\theta_{s}\cos\varphi_{s}$, $k_{y}=k\sin\theta_{i}\sin\varphi$, $k_{sy}=k\sin\theta_{s}\cos\varphi_{s}$, θ is the scattering angle, φ and $\varphi_{s}$ represent the incident azimuth and scattering azimuth, respectively, and $F_{pq}$ and $f_{pq}$ are Fresnel reflection coefficients ($\Gamma_{pp}$).

The AIEM model redefines the Fresnel reflection coefficient ($\Gamma_{pp}$) to apply to any roughness condition. The relationship is as follows:

(8) $$\Gamma_{pp}=\Gamma_{pp}\left(\theta \right)+\left[\Gamma \left(0\right)-\Gamma_{pp}\left(\theta \right)\right]\left(1-\frac{S_{p}}{S_{0}}\right)$$

The equation terms are defined as follows:

(9) $$S_{0}=\frac{1}{{\left|1+\frac{8R_{p}\left(0\right)}{F_{p}\cos\theta }\right|}^{2}}$$

(10) $$S_{p}=\frac{{\left|F_{p}\right|}^{2}\sum_{n=1}^{\infty }\frac{{\left(ks\cdot \cos\theta \right)}^{2n}}{n!}\cdot W^{\left(n\right)}\left(2k\sin\theta \right)}{\sum_{n=1}^{\infty }\frac{{\left(ks\cdot \cos\theta \right)}^{2n}}{n!}\left|F_{p}+\frac{2^{n+2}\cdot R_{p}\left(0\right)}{\exp{\left(ks\cdot \cos\theta \right)}^{2}\cdot \cos\theta }\right|\cdot W^{\left(n\right)}\left(2k\sin\theta \right)}$$

(11) $$F_{p}=8\Gamma_{p}^{2}\left(0\right)\cdot \sin^{2}\theta \cdot \left(\frac{\cos\theta +\sqrt{\varepsilon -\sin^{2}\theta }}{\cos\theta -\sqrt{\varepsilon -\sin^{2}\theta }}\right)$$

In the equation above, ε is the dielectric constant of soil and the relationship between ε and soil moisture is as follows:

(12) $$\varepsilon^{\alpha }≅1+\frac{\rho_{b}}{\rho_{s}}\left(\varepsilon_{s}^{\alpha }-1\right)-M_{v}^{\beta }\varepsilon_{fw}^{\alpha }-M_{V}$$

In Equation (12), $\rho_{b}$ is the soil bulk density, $\rho_{s}$ is the soil density, $\varepsilon_{s}$ is the soil solid dielectric constant (approximately 4.7), $\varepsilon_{fw}$ is the pure water dielectric constant, and α and β are soil texture constants.

## 4. Results and Discussion

### 4.1. Analysis of Responses of the Backscattering Coefficient and Surface Parameters

When applying the AIEM model, the input parameters include the incidence angle, dielectric constant, root mean square height, and correlation length. For a certain radar system, the incidence angle can be obtained from the radar system header file; in practical applications, the relationships between the backscattering coefficient and the last three parameters are mainly considered.

Physical and chemical analyses of the soil samples collected in the study area revealed that the soil texture type of the area was mainly loam, and the remaining few types of textures were clay and sand. According to the measured soil texture average and the soil type classification map, the sandy clay loam is the soil type of the whole study area, where in the mass fractions of sand, powder and clay are 60%, 20% and 20%, respectively. The average soil bulk density in the target area was 1.4 g/cm^3^, and the dielectric constant of 5%–50% soil moisture was calculated in 3% step size to prepare the AIEM model to simulate the backscattering coefficient. The reference incidence angle is set as the centre incidence angle of the Sentinel-1 data, which is approximately 39°.

#### 4.1.1. Analysis of Relationship Between the Backscattering Coefficient and Soil Moisture

**Figure 3:**
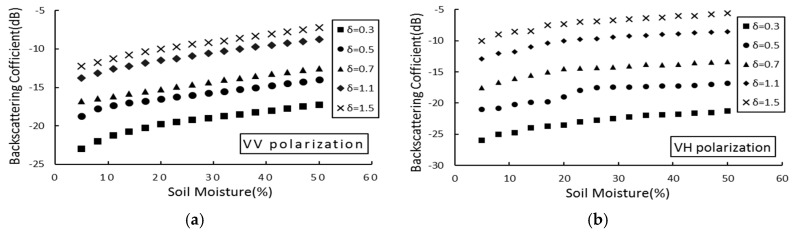
Effect of soil moisture change on the backscattering coefficient. θ = 39 °, l = 15 cm. (**a**) VV polarization; (**b**) VH polarization.

In the C-band (5.33 GHz), the AIEM model was used to simulate the relationship between five (0.3 cm, 0.5 cm, 0.7 cm, 1.1 cm, 1.5 cm) root mean square height backscatter coefficients and soil moisture, under the condition of an incident angle (θ) of 39 °, correlation length (l) of 15 cm, and two (VV and VH) polarization modes. The results are shown in Figure 3.

**Figure 4:**
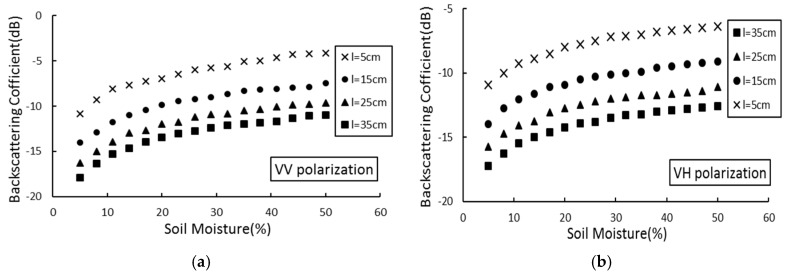
Effect of soil moisture change on the backscattering coefficient. θ = 39°; δ = 0.5 cm. (**a**) VV polarization; (**b**) VH polarization.

In the C-band (5.33 GHz), the AIEM model was used to simulate the relationship between four (5 cm, 15 cm, 25 cm, 35 cm) correlation lengths backscatter coefficients and soil moisture, under the condition of an incident angle (*θ*) of 39°, root mean square height (δ) of 0.5 cm, and two (VV and VH) polarization modes. The results are shown in Figure 4.

As shown in Figure 3 and Figure 4, there is a significant nonlinear relationship between soil moisture and the backscattering coefficient. As the soil moisture increases, the backscattering coefficient increases continuously. Figure 3 shows that in the case of the same soil moisture, as the height of the root mean square increases, the backscattering coefficient also increases gradually. When the root mean square height is 1.5 cm, the backscattering coefficient reaches the minimum; Figure 4 shows that in the case of the same soil moisture, with increasing of the correlation length, the backscattering coefficient gradually decreases. When the correlation length is 35 cm, the backscattering coefficient reaches the minimum.

**Figure 5:**
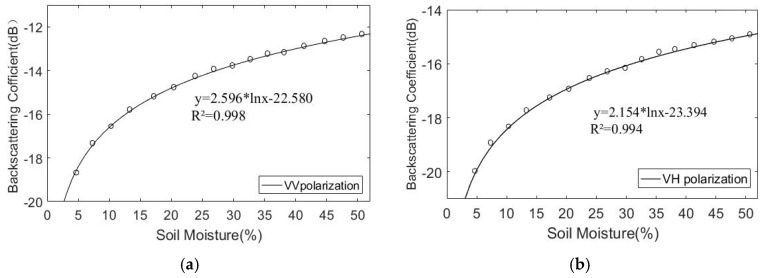
Regression functions between soil moisture and the backscattering coefficient. (**a**) VV polarization; (**b**) VH polarization.

Figure 3 and Figure 4 show that the logarithmic relationship between soil moisture and the backscattering coefficient remains constant at different root mean square heights and correlation lengths. Simulation analysis of soil moisture and backscattering coefficient was conducted. Figure 5 shows the functional expression between the backscattering coefficient and the soil moisture in two polarization modes (VV and VH), under the condition of the C-band, incident angle of 39°, root mean square height of 0.5 cm, and correlation length of 15 cm.

From the response curve, it can be concluded that the backscattering coefficient and soil moisture show a good logarithmic relationship in both polarization modes, this relationship can be expressed by the following equation:

(13) $$\sigma_{pq}^{0}=A_{pq}\ln(m_{v})+f\left(\mathrm{roughness}\right)$$

The coefficient $A_{pq}$ in the equation has no relationship with the root mean square height and the correlation length.

We conclude that the specific expression of the roughness term should be determined when using the equation to retrieve soil moisture, and the inversion results of soil moisture can be obtained.

#### 4.1.2. Analysis of the Relationship Between Backscattering Coefficient and Surface Roughness

Researchers have explored the relationship between the correlation length and the root mean square height and established an empirical equation between the root mean square height and correlation length [45,46,47]. Shi et al. [48] combined the root mean square height and correlation length into one parameter with a combined parameter expression of $S_{R}=\left(k\delta \right)W$. Oh et al. [14,49] adopted the combined parameter δ/l to characterize the degree of surface roughness. Zribi et al. [50] used the combined roughness parameter $Z_{s}=\delta^{2}/l$ to invert the surface parameters, and these combination parameters achieved good results. In this study, combined parameters were used to represent the surface roughness because it can better characterize the roughness of the natural surface.

**Figure 6:**
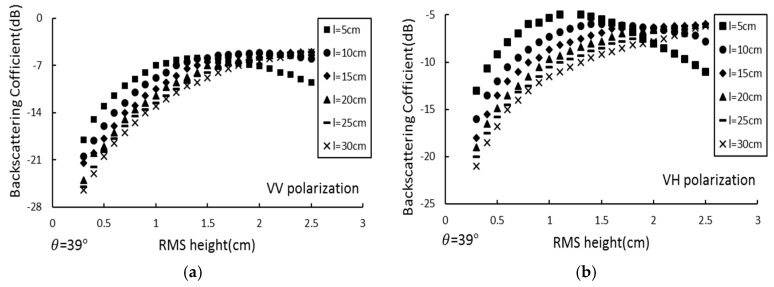
Effect of different incidence angle on the RMS height changes response of the backscattering coefficient. (**a**) VV polarization; (**b**) VH polarization.

For an incident angle of θ = 39°, the relationship between the C-band (5.33 GHz) backscattering coefficient and the surface roughness was analyzed. Figure 6 shows the response between the backscattering coefficient and the root mean square height under different correlation lengths (6, 10, 15, 20, 25 and 30 cm) and two polarization modes (VV and VH).

**Figure 7:**
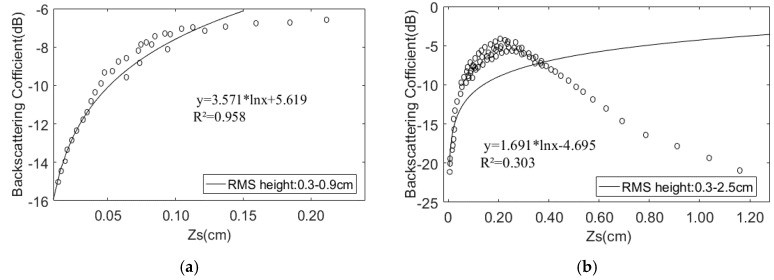
Reletionship between the backscattering coefficient and *Z_s_*. (**a**) δ: 0.3–0.9 cm; (**b**) δ: 0.9–2.5 cm.

Figure 6 shows that for different correlation lengths, regardless of the VV or VH polarization conditions, the response relationship between the backscattering coefficient and the root mean square height increases with increasing RMS height; over 0.9 cm, this trend tends to saturate or decrease as the root mean square height increases. The analysis indicates that this trend is not related to the correlation length. The establishment of this relationship is the basis for retrieving the surface roughness using the radar backscatter coefficient. Due to this response relationship, this paper only discusses the response of the combined roughness parameter *Z_s_* to the backscattering coefficient from 0.3–0.9 cm and 0.3–2.5 cm for HH polarization (Figure 7).

Figure 7 shows that when the root mean square height is from 0.3–0.9 cm, the correlation coefficient between the combined roughness parameter *Z_s_* and the backscattering coefficient is 0.958, and the correlation between the two is in the range of 0.3–2.5 cm. The coefficient is only 0.303. This shows that under the C-band condition, the root mean square height is 0.3–0.9 cm, and there is a good logarithmic relationship between the combined roughness parameter and the backscattering coefficient. When the local table roughness range is large, it cannot be simple. The logarithmic relationship is used to describe the relationship between the combined roughness parameter and the backscatter coefficient. The analysis shows that the logarithmic model is suitable for areas with low surface roughness, which is consistent with the actual situation of the study area.

Based on the above analysis, the relationship between the backscattering coefficient $\sigma^{0}$ and the combined roughness parameter *Z_s_* can be expressed as:

(14) $$\sigma_{pq}^{0}=B_{pq}\ln(Z_{s})+f\left(m_{v}\right)$$

The coefficient *B* in the equation is independent of the water content.

### 4.2. Constructing the Soil Moisture Inversion Model 

The relationship between the soil moisture and backscatter coefficient is:

(15) $$\sigma_{pq}^{0}=A_{pq}\ln(m_{v})+f\left(\mathrm{roughness}\right)$$

The coefficient A in the equation is independent of the surface roughness under certain incident angle conditions. The relationship between the backscattering coefficient and the surface combination roughness parameter is as follows:

(16) $$\sigma_{pq}^{0}=B_{pq}\ln(Z_{s})+f\left(m_{v}\right)$$

From these two equations, the relationship between backscattering coefficient and soil moisture and surface roughness can be given by:

(17) $$\sigma_{pq}^{0}=A_{pq}\left(\theta \right)\ln(m_{v})+B_{pq}\left(\theta \right)\ln(Z_{S})+C_{pq}\left(\theta \right)$$

The coefficients *A*(*θ*), *B(θ),* and *C*(*θ*) in the equation are only related to the magnitude of the incident angle and are a function of the incidence angle *θ*. Therefore, to reproduce the soil moisture, the exact expressions of the coefficients *A*(*θ*), *B*(*θ*), and *C*(*θ*) should be determined. To determine the expressions of these three coefficients, the AIEM model was used to simulate incident angles *θ* of 11°–61°, the soil moisture mv was 5%–50%, the root mean square height was 0.3–0.9 cm, and the correlation length is 5–30 cm. The step angle of the incident angle was 2°, the step size of the soil moisture was 3%, the step height of the root mean square was 0.1 cm, and the step length of the relevant length was 3 cm. Using the least squares method to perform nonlinear fitting, the specific values of *A*(*θ*), *B*(*θ*), *C*(*θ*) under each incident angle condition were calculated (Table 3 and Table 4) and a nonlinear regression was performed on these data [51,52] to obtain an expression of *A*(*θ*), *B*(*θ*), *C*(*θ*) with respect to the incidence angle.

According to the simulated relationship, the expressions of *A*(*θ*), *B*(*θ*), *C*(*θ*) under VV polarization are:

(18) $$A=2.202\sin^{3}\theta -2.101\sin^{2}\theta +1.765\sin\theta +2.195$$

(19) $$B=6.491\;\sin^{3}\theta -10.236\;\sin^{2}\theta +1.580\;\sin\theta +1.58$$

(20) $$C=-21.940\;\sin^{3}\theta +42.230\;\sin^{2}\theta -53.251\;\sin\theta +20.591$$

According to the simulated relationship, the expressions of *A*(*θ*), *B*(*θ*), *C*(*θ*) under VH polarization are:

(21) $$A=2.441\;\sin^{3}\theta -3.337\;\sin^{2}\theta +2.975\;\sin\theta +1.970$$

(22) $$B=5.754\;\sin^{3}\theta -7.595\;\sin^{2}\theta +4.813\;\sin\theta +1.811$$

(23) $$C=-22.839\;\sin^{3}\theta +67.403\;\sin^{2}\theta -60.539\;\sin\theta +21.390$$

Therefore, as long as the incidence angle information can be obtained from the radar images, the soil moisture inversion model can be constructed.

### 4.3. Removing the Vegetation Effect

Vegetation Water Content (*vwc*) is the moisture content of vegetation per unit area, which is usually obtained by drying vegetation in field investigations. To dynamically monitor vegetation moisture content in a wide range of underlying surfaces, scholars have proposed many remote sensing parameters [53] including the NDVI, water index (WI), normalized difference water index (NDWI), and simple ratio index [54,55,56]. Among those, the best simulated effect is the Normalized Difference Moisture Index (NDMI). In the Ugan-Kuqa River Delta Oasis, scholars have used optical remote sensing imagery to invert the water content of the plant. Nigra [41] used this model to invert the vegetation water content of the Ugan-Kuqa River Delta Oasis. The inversion accuracy was in line with the actual situation, indicating that the model has good universality in the Ugan-Kuqa River Delta Oasis. The vegetation water content extraction model is as follows [41]:

NDMI = (NIR − MIR)/(NIR + MIR)(24)

(25) vwc=2.15×NDMI+0.32

**Figure 8:**
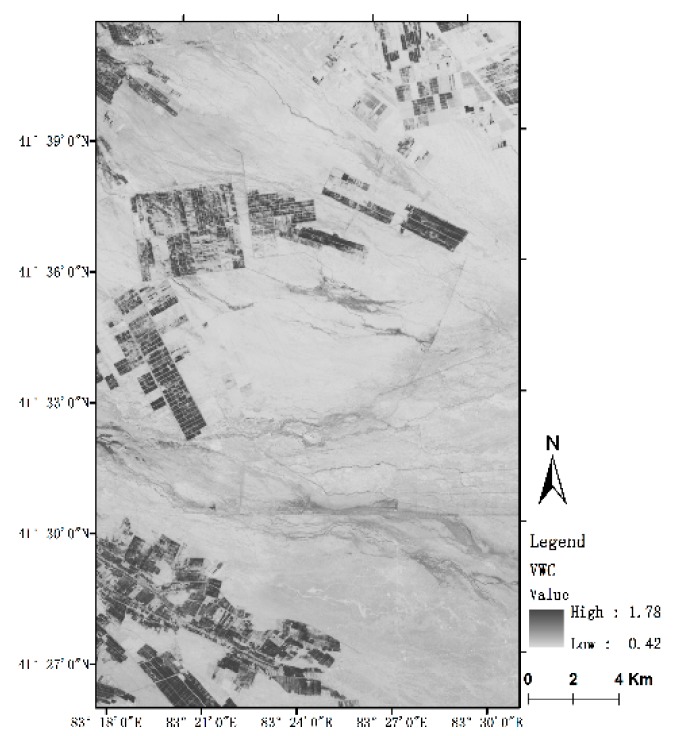
Spatial distribution of the vegetation water content.

Based on the Landsat-8 OLI data, the above equations were used to invert the vegetation water content information of the target area (Figure 8). Figure 8 shows that, in the study area of the Oasis-Desert Ecotone, where the area with larger water content are mainly concentrated in farmland and marginal oases. The water content of vegetation is more than 1 kg/m^2^, the crops have better upswing.The water content of vegetation outside the oasis is lower, most of which are less than 0.5 kg/m^2^, Most of the vegetation in this area is halophytes and shorter shrubs. From the periphery of the oasis to the desert area, the water content of the vegetation is significantly reduced, and the water content of the farmland in the interlaced zone is significantly lower than that inside the oasis, indicating that the vegetation has poor potential or low vegetation coverage, consistent with the actual situation.

**Figure 9:**
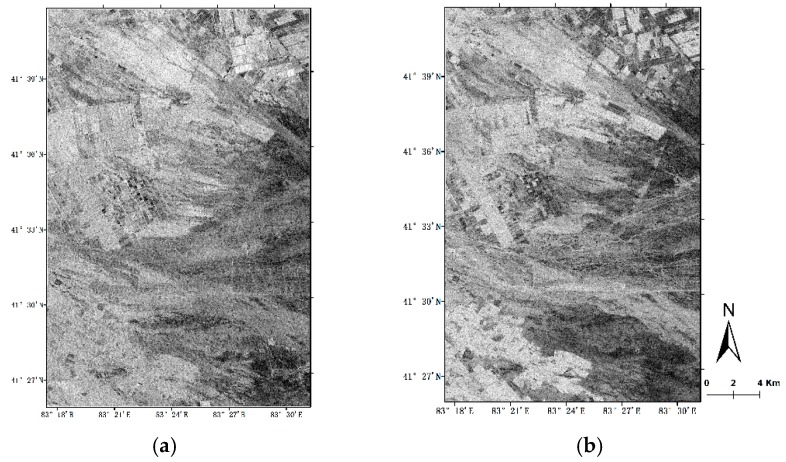
Remove vegetation effect. (**a**) VV Polarization; (**b**) VH Polarization.

To remove the influence of vegetation water content on the radar backscattering coefficient and ensure the radar data is sufficient, the resolution of the radar image resampling and oblique ground distance was 20 m, and the Landsat OLI data was resampled to 20 m. Different data source images have the same spatial resolution. Using Equation (5), the backscattering coefficients of the two polarization modes after removing the vegetation effect are obtained, and the results are shown in Figure 9.

### 4.4. Remote Sensing Inversion of the Spatial Distribution of Soil Moisture

Reference to Equations (18)–(23) and according to the incidence angle data extracted from the radar image data header file, expressions in both polarization modes can be obtained:

(26) $$\sigma_{vv}^{0}=2.934\;\ln(m_{v})+0.339\;\ln(Z_{S})-0.237$$

(27) $$\sigma_{vh}^{0}=3.042\;\ln(m_{v})+3.972\;\ln(Z_{S})+4.524$$

Mathematical operations were performed on the above equations to obtain the expressions for surface roughness and soil moisture:

(28) $$\mathrm{Mv}=\exp\frac{\left(3.972\cdot \sigma_{vv}^{0}-0.339\cdot \sigma_{vh}^{0}+2.475\right)}{10.623}$$

**Figure 10:**
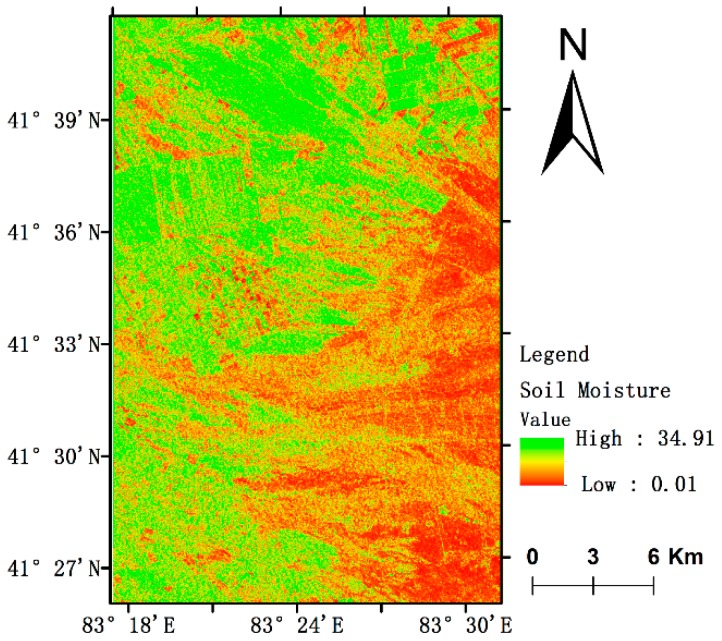
The spatial distribution information of soil moisture.

Referring to Equation (28) and based on the Sentinel-1 microwave data, the spatial distribution information of soil moisture in the study area were obtained by inversion (Figure 10), and their error metrics, i.e., bias, standard error (RMSE), and slope are listed in Table 5.

In general, the accuracy of the soil moisture estimation is promising. The integrated bias, RMSE and slope are 0.039, 0.97, and 0.8894, respectively. A positive bias indicates that the retrieved soil moisture is systematically overestimated compared with the in situ measurements. The RMSE, which represent the absolute errors between the observations and simulations.

Figure 10 shows that the soil water content is high in the northwest and low in the southeast. The high soil water content is mainly concentrated in the edge of the oasis, in some areas the water content can reach 30%. The main reason is the vegetation coverage, which has a certain water storage capacity for water and there is also the impact of crop irrigation; the farther away from the oasis region, the lower the soil moisture content be, and the soil moisture content in some areas can less than 1%, mainly due to the low vegetation coverage, serious desertification, and low soil water holding capacity and the evaporation is relatively high, leading to extreme drought.

**Figure 11:**
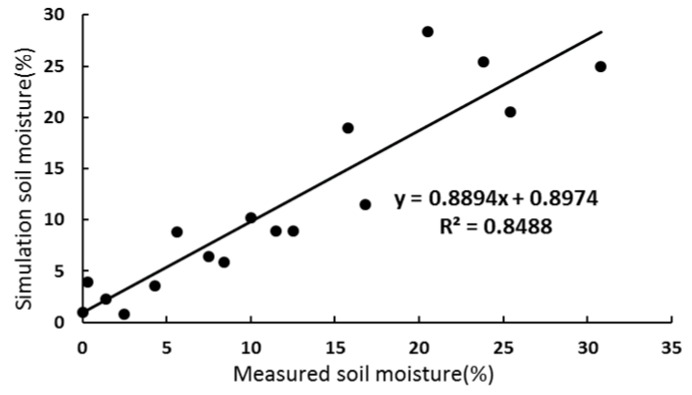
The correlation of the soil moisture data between simulation and measured values.

In order to verify the accuracy of the soil moisture model constructed in this paper, the Sentinel-1 radar image simulation values were correlated with the 0–10 cm surface soil measured values obtained from the field sampling experiments in the same study area. The results are shown in Figure 11.

Figure 11 shows that the correlation coefficient between the measured soil moisture and inversion of soil moisture content reached 0.8488, which was significantly correlated, indicating that the soil moisture inversion model established in this study can meet the requirements in the study area. Thus, AIEM model is suitable for soil moisture inversion in the Ugan-Kuqa River Delta Oasis.

## 5. Conclusions

This study mainly investigates the relationship between the radar backscattering coefficient and system parameters (polarization mode, incidence angle) and surface parameters (soil moisture content, surface roughness) under multiband, multi-angle and multipolar conditions. The relationships between the radar backscattering coefficient and soil moisture, the root mean square height and combined roughness parameters were analyzed, the optimal parameters to invert the surface parameters were selected, and the salinization suitable for arid areas can be proposed using an empirical model for soil radar inversion of surface soil moisture. Based on this study, the following conclusions can be drawn:

(1) Under different root mean square heights and correlation lengths, the backscattering coefficients of both VV and VH polarization modes have a good logarithmic relationship with soil moisture, and the relationship remains unchanged. Under the given incident angle, the backscattering coefficient is independent of the surface roughness and is a function of the soil moisture content.
(2) According to the actual situation of the study area, the surface roughness parameters are used to characterize the surface roughness, and the relationship between the combined roughness and the backscatter coefficient was analyzed. Under the smooth surface (root mean square height 0.3–0.9 cm), there is a dominant logarithmic relationship model between the two; under the rough surface (root mean square height is 0.9–2.5 cm), the logarithmic relationship cannot simply be used to describe the relationship between the two, and it is necessary to determine a suitable model.
(3) A quantitative remote sensing inversion model of soil moisture under the dual-polarization condition was established based on the logarithmic relationship. The model is based on the theoretical AIEM model and can better reflect the relationship between the backscattering coefficient and soil moisture. Compared with the traditional empirical model, it is less restricted by the region and has better universality. The correlation between the model simulation results and the measured values is very strong, indicating that the model established in this study can be used for the inversion of surface soil moisture.

The AIEM model does not consider the influence of vegetation on microwave backscattering, and the surface scattering characteristics of high vegetation cover and crop growth season cannot be accurately described. Therefore, the empirical model established in this study can only be used for bare ground surface and surface conditions in the early stage of crop growth. Future research should analyze the microwave scattering characteristics of vegetation cover surfaces, eliminate the influence of vegetation on the backscatter coefficient, and establish suitable vegetation cover for the soil moisture inversion model of the surface. Research should also focus on the use of radar data from different bands and various time periods for soil moisture inversion, improve the accuracy of radar data for soil moisture inversion under different roughness conditions, and conduct soil moisture inversion model and soil-vegetation-atmospheric model coupling studies soil moisture profiles to improve radar reversal of soil moisture.

## Figures and Tables

**Table 1:** Features of the land cover classes selected for classification.

Class	Sites	Description
Farm	26	Planting crops
Wetland	5	Almost all vegetation is shrubs with a high vegetation coverage
Bare soil	24	No vegetation cover
Grass	11	Saline vegetation, shrub
Salinated land	28	No vegetation cover, but there are salt shells on the surface

**Table 2:** Vegetation parameters in a semi empirical model.

Parameter	All Vegetation	Grazing Land	Crop	Grass
A	0.0012	0.0009	0.0018	0.0014
B	0.091	0.032	0.138	0.084

**Table 3:** The coefficients of different incidence angles of VV polarization.

*θ*	*A*(*θ*)	*B*(*θ*)	*C*(*θ*)	Standard Deviation	*R* ^2^	*θ*	*A*(*θ*)	*B*(*θ*)	*C*(*θ*)	Standard Deviation	*R* ^2^
11	2.402	2.455	12.766	0.610	0.832	37	3.054	3.196	4.526	0.523	0.920
13	2.479	2.497	11.789	0.656	0.828	39	3.096	3.235	4.432	0.537	0.911
15	2.513	2.601	10.652	0.662	0.821	41	3.143	3.289	4.315	0.458	0.929
17	2.564	2.689	9..632	0.601	0.839	43	3.187	3.355	4.223	0.465	0.931
19	2.603	2.742	8.698	0.678	0.825	45	3.232	3.416	4.136	0.399	0.936
21	2.678	2.795	7.923	0.598	0.898	47	3.297	3.496	4.045	0.425	0.929
23	2.741	2.846	7.212	0.621	0.830	49	3.347	3.562	4.212	0.371	0.938
25	2.796	2.899	6.625	0.635	0.828	51	3.395	3.628	4.287	0.457	0.929
27	2.846	2.932	5.945	0.641	0.824	53	3.438	3.701	4.378	0.565	0.901
29	2.898	2.998	5.321	0.580	0.902	55	3.486	3.789	4.567	0.498	0.916
31	2.938	3.021	4.852	0.601	0.839	57	3.531	3.869	4.579	0.465	0.923
33	2.996	3.079	4.765	0.633	0.829	59	3.597	3.945	4.583	0.441	0.925
35	3.012	3.148	4.679	0.574	0.900	61	3.621	4.012	4.635	0.432	0.931

**Table 4:** The coefficients of different incidence angles of VH polarization.

*θ*	*A*(*θ*)	*B*(*θ*)	*C*(*θ*)	Standard Deviation	*R* ^2^	*θ*	*A*(*θ*)	*B*(*θ*)	*C*(*θ*)	Standard Deviation	*R* ^2^
11	2.474	2.405	12.380	0.607	0.831	37	2.954	3.387	1.103	0.526	0.919
13	2.492	2.604	11.565	0.678	0.825	39	2.982	3.416	0.568	0.539	0.912
15	2.512	2.796	10.638	0.659	0.827	41	3.012	3.469	-0.053	0.465	0.926
17	2.537	2.832	9.658	0.600	0.852	43	3.073	3.506	-0.465	0.403	0.932
19	2.578	2.901	8.065	0.687	0.815	45	3.146	3.559	-1.011	0.398	0.936
21	2.602	2.985	7.049	0.596	0.895	47	3.201	3.625	-1.368	0.423	0.930
23	2.691	3.024	6.123	0.632	0.829	49	3.267	3.687	-1.769	0.369	0.942
25	2.725	3.068	5.326	0.645	0.823	51	3.301	3.712	-2.145	0.354	0.951
27	2.767	3.102	4.505	0.651	0.825	53	3.364	3.765	-2.687	0.312	0.962
29	2.802	3.159	3.724	0.598	0.898	55	3.412	3.829	-3.269	0.201	0.989
31	2.842	3.211	3.012	0.603	0.836	57	3.478	3.897	-3.755	0.102	0.995
33	2.897	3.275	2.225	0.643	0.823	59	3.521	3.946	-4.052	0.326	0.960
35	2.921	3.326	1.687	0.586	0.901	61	3.586	4.012	-4.368	0.128	0.992

**Table 5:** Statistical metrics between in situ and simulation soil moisture.

Model	Bias	RMSE	Slope
AIEM	0.039	0.97	0.8894
